# Non-traumatic Spontaneous Paraplegia Secondary to Thoracic Disc Herniations in the Setting of Tobacco Abuse and COVID-19: A Case Report and Literature Review

**DOI:** 10.7759/cureus.31544

**Published:** 2022-11-15

**Authors:** Eric Whitney, John W Kiessling, Louis Reier, Kyle Hamann, Mohammad Arshad, Ajay Ramnot, James Fowler

**Affiliations:** 1 Neurosurgery, Desert Regional Medical Center, Palm Springs, USA; 2 Surgery, Midwestern University Chicago College of Osteopathic Medicine, Downers Grove, USA; 3 Neurological Surgery, Hartford Hospital, Hartford, USA

**Keywords:** acute paraplegia, surgery spine, tobacco abuse, covid 19, thoracic disc herniation

## Abstract

Thoracic disc herniations (TDHs) are very rare. While most common in the setting of trauma, other etiologies have been documented. Here, we present a case of spontaneous TDHs in the setting of tobacco abuse and coronavirus disease 2019 (COVID-19) causing acute paraplegia. We review spontaneous TDHs, associated risk factors, and the possible role of COVID-19 in the pathophysiology.

## Introduction

Disc herniations are well-documented pathologies affecting roughly 50 per 100,000 people, most commonly involving the lumbar vertebrae, with thoracic disc herniations (TDHs) being a small subset [1]. TDHs comprise 0.5% to 4.5% of symptomatic disc herniations and only 0.15% to 1.8% of all surgically treated herniations [2].

The low incidence of TDHs in relation to other anatomic regions is related to the relative immobility and lower load-bearing demands of the thoracic spine. Both the coronal orientation of the facet joints and anchoring ribs provide stability with less repetitive trauma from physiological motion. Repetitive trauma of the more mobile cervical and lumbar regions contributes to degenerative changes over time. The resulting damage of the outer annulus ﬁbrosis manifests in disc herniations among others. Furthermore, the thoracic spine has less weight-bearing load compared to the lumbar segments [2]. Within the thoracic spine, the lower thoracic segments bear the most axial load, and as a result, the most common levels for TDHs occur at T8-T12 (roughly 75% of the time). Large body habitus, as seen in our patient, contributes to increased load on all spinal levels, compounded at the lower thoracic levels and lumbar spine. The T11-T12 disc is the most common level for TDHs secondary to the increased axial load and increased mobility of the segment as it transitions to the lumbar spine. Additionally, given that T11-T12 is a transitional zone, the posterior longitudinal ligament has more laxity at this level compared to the rest of the thoracic spine, which is believed to further contribute to the higher incidence of TDHs occurring at this level [1].

## Case presentation

A 35-year-old male with a medical history of obesity, tobacco abuse, and recent diagnoses of coronavirus disease 2019 (COVID-19) presented to our hospital from an outside facility with a chief complaint of bilateral lower extremity weakness, onset eight hours prior to his arrival. The patient had no trauma or inciting events to bring upon this weakness. He noticed signiﬁcant back spasms when he sat down to play video games earlier that morning, a few hours prior to the symptom onset. By the time he arrived at our hospital's emergency department, he was completely paraplegic in bilateral lower extremities and had an episode of urinary incontinence. Pertinent physical exam ﬁndings included 0/5 strength throughout bilateral lower extremities, complete loss of sensation from thoracic (T)11 dermatome down, and no rectal tone but intact bulbocavernosus reﬂex. His exam was consistent with a T11 American Spinal Injury Association (ASIA) grade A spinal cord injury (SCI).

**Figure 1:**
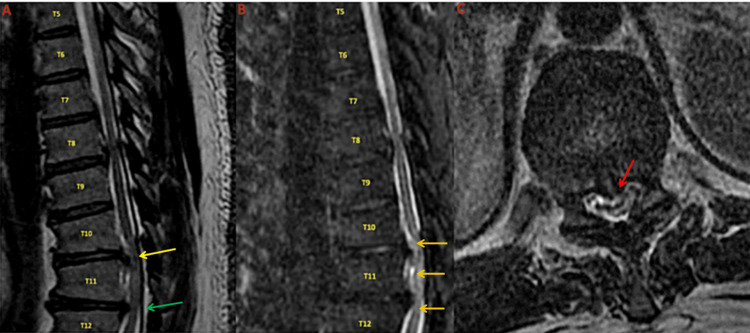
MRI thoracic spine A. MRI T2 weighted image in the sagittal plane showing multilevel herniated discs. The largest herniated discs and the ones responsible for the patient's acute symptoms are at T10-T11 (yellow arrow) and T11-T12 (green arrow). B. MRI STIR sequence in the sagittal plane demonstrating multiple disc herniations. The spinal cord is hyperintense from T10-T12 compatible with edema or ischemia in the acute setting (yellow arrows). C. MRI T2 weighted image in the axial plane at the level of T10-T11, demonstrating a large posterior central disc protrusion causing signiﬁcant cord compression (red arrow). MRI: Magnetic resonance imaging; STIR: Short T1 inversion recovery; T: thoracic

Emergent magnetic resonance imaging (MRI) demonstrated calciﬁed and herniated thoracic discs at T10-T11 and T11-T12 with signiﬁcant central canal stenosis and compression of his spinal cord (as seen in Figure 1). The patient underwent emergent decompressive discectomy at the affected levels with T10-T12 posterior instrumented fusion, within 12 hours of the symptom onset. The patient did not have any further decline in his symptoms after surgery; however, when re-evaluated two weeks postoperatively while in acute rehab, he still had not regained any function.

Images

MRI thoracic spine without contrast was obtained and demonstrated a large (5 mm) posterior disc protrusion at the T10-T11 level, in the setting of a congenitally narrowed thoracic spinal canal from short pedicles. An increased central cord signal compatible with edema or ischemia in the acute setting, as well as severe cord compression, was seen. Additionally, there was mild central, posterior disc protrusion superimposed on a generalized disc bulge at the T11-T12 level causing severe central canal stenosis and a milder degree of cord compression. At the T7-T8 and T8-T9 levels, both had paracentral posterior disc protrusions causing moderate to severe central canal stenosis and a mild degree of cord compression with ﬂattening upon the ventral cord contour.

In summary, pertinent MRI ﬁndings include congenital stenosis of the thoracic spine with herniated discs at T10-T11 and T11-T12 causing cord compression (as seen in Figure 1) correlating clinically with the patient's exam (T11 ASIA-A SCI).

## Discussion

While TDHs are most commonly present in the setting of trauma (either external trauma or microtrauma in the setting of physiological movement making up 49% of all causes of TDHs [3]), a subset of TDHs can be seen with other associated conditions such as Scheuermann’s disease in the pediatric population, hereditary connective tissue disorders such as Ehlers-Danlos syndromes or Marfan syndrome, smoking, congenital stenosis, obesity, and those with sedentary lifestyles [4]. Rarely, spontaneous cases have been reported such as a case where two brothers aged 32 and 34 both had TDHs with no other predetermined risk factors suggesting an underlying genetic predisposition [4]. Our patient had no inciting trauma, but tobacco and underlying congenital stenosis were identiﬁed as risk factors that likely contributed to disc degeneration and acute presentation. There are two main theories as to how tobacco use may lead to increased intervertebral disc degeneration. The ﬁrst accentuates the role of anoxia seen in vascular disease, in which smoking is known to precipitate. The effect of reduced vertebral blood ﬂow after the administration of nicotine has been studied in animal models [5]. The second theory proposes the speciﬁc effect that the introduction of nicotine has on the disc directly. Nicotine has been shown to increase the levels of IL-1B, which directly increases the amount of metalloproteinases in the system [5]. These metalloproteinases directly degrade the disc matrix. Battie et al. found a decrease of 18% disc height mean reported between smoking and non-smoking groups [6]. Perhaps holding more statistical power, the study by Mattila et al. followed almost 60,000 adolescents over an 11-year period and sought to identify risk factors for lumbar disc herniations. The results of the study showed that when referring to male adolescents, daily cigarette smoking was associated with the highest correlation with the need for lumbar discectomy [7].

Congenital spinal stenosis is a phenomenon where the individual is born with a spinal canal that is smaller than anticipated, given the dimensions of the vertebral body. In the instance of a disc herniation, the decreased canal volume increases the possibility that the spinal cord will be compressed, leading to an increased frequency in symptom presentation such as pain, paresthesia, and motor deﬁcits. Although this phenomenon has not been studied in the thoracic region speciﬁcally, Wiley et al. found that adolescent patients with an identiﬁed lumbar spinal stenosis were 18.8 times more likely to need surgical decompression following disc herniations. The assumption made in the study is that since the population was adolescents, any spinal stenosis found was congenital [8].

Seeing as though the severe acute respiratory syndrome coronavirus 2 (SARS-CoV-2) is still new, there is a lack of deﬁnitive research evaluating all the possible sequelae the virus might have. That being said, the potential effect of the progression of degenerative spinal diseases has been studied. Guadarrama-Ortiz et al. explored the exacerbated neurological symptoms of a 73-year-old male with a chronic subclinical cervical spondylotic myelopathy. They proposed that the expression of angiotensin converting enzyme 2, which is the spike receptor of the SARS-CoV-2 virus, is overregulated by inﬂammatory signals [9]. This leads to increased permeability of the blood-spinal cord barrier which can allow for more inﬂammatory inﬁltration. It is possible that the enhanced viral inﬂammatory response exacerbated the subclinical inﬂammation that was already present leading to the clinical presentation due to compressive myelopathy [9].

## Conclusions

TDH continues to be a rare pathology and, when seen, is most commonly due to trauma. Here, we have presented a 35-year-old male who suffered a non-traumatic spontaneous disc herniation leading to severe neurological injury, and his main risk factors were obesity, congenital stenosis, smoking history, and recent COVID-19 infection. The majority of these risk factors are fairly prevalent in the population, but COVID-19 represents a new variable that may potentially place these people at further increased risk for atraumatic spontaneous TDHs. More research and reviews are necessary to delineate what may or may not be truly contributing risk factors. However, the introduction of COVID-19 into our society and the discovery of multiple systemic effects due to infection may contribute to a potential increase in the incidence of these types of TDHs, which may have devastating clinical consequences.
